# Hepcidin Is Involved in Iron Regulation in the Ischemic Brain

**DOI:** 10.1371/journal.pone.0025324

**Published:** 2011-09-21

**Authors:** Hui Ding, Cai-Zhen Yan, Honglian Shi, Ya-Shuo Zhao, Shi-Yang Chang, Peng Yu, Wen-Shuang Wu, Chen-Yang Zhao, Yan-Zhong Chang, Xiang-Lin Duan

**Affiliations:** 1 Laboratory of Molecular Iron Metabolism, College of Life Science, Hebei Normal University, Shijiazhuang, China; 2 Department of Pharmacology, Hebei Medical University, Shijiazhuang, China; 3 Department of Pharmacology and Toxicology, School of Pharmacy, University of Kansas, Lawrence, Kansas, United States of America; The Mental Health Research Institute of Victoria, Australia

## Abstract

Oxidative stress plays an important role in neuronal injuries caused by cerebral ischemia. It is well established that free iron increases significantly during ischemia and is responsible for oxidative damage in the brain. However, the mechanism of this ischemia-induced increase in iron is not completely understood. In this report, the middle cerebral artery occlusion (MCAO) rat model was performed and the mechanism of iron accumulation in cerebral ischemia-reperfusion was studied. The expression of L-ferritin was significantly increased in the cerebral cortex, hippocampus, and striatum on the ischemic side, whereas H-ferritin was reduced in the striatum and increased in the cerebral cortex and hippocampus. The expression level of the iron-export protein ferroportin1 (FPN1) significantly decreased, while the expression of transferrin receptor 1 (TfR1) was increased. In order to elucidate the mechanisms of FPN1 regulation, we studied the expression of the key regulator of FPN1, hepcidin. We observed that the hepcidin level was significantly elevated in the ischemic side of the brain. Knockdown hepcidin repressed the increasing of L-ferritin and decreasing of FPN1 invoked by ischemia-reperfusion. The results indicate that hepcidin is an important contributor to iron overload in cerebral ischemia. Furthermore, our results demonstrated that the levels of hypoxia-inducible factor-1α (HIF-1α) were significantly higher in the cerebral cortex, hippocampus and striatum on the ischemic side; therefore, the HIF-1α-mediated TfR1 expression may be another contributor to the iron overload in the ischemia-reperfusion brain.

## Introduction

Many mechanisms are involved in ischemia-induced brain injuries, such as oxidative stress [1], [2], increased intracellular calcium concentration [3], [4], [5], inflammation [6], [7], and elevated excitatory amino acids [8]. Iron, the most abundant trace metal in the brain, is also believed to play a critical role in neuronal injuries caused by oxidative stress in ischemia, although the exact mechanism is not understood. Increased levels of free iron and ferritin have been observed in ischemic brain [9], [10]. Free iron is a major generator of reactive oxygen species, which are capable of damaging biological molecules such as lipids, carbohydrates, proteins, and nucleic acids [11], [12]. Furthermore, during hypoxia, free iron appears to accelerate intracellular free radical formation and lipid peroxidation, causing subsequent neuronal injury and death [13], [14]. The damaging role of free iron in the ischemic brain is underscored by the beneficial effects of the iron chelator desferrioxamine (DFO) [13], [15], [16].

Under physiological conditions, cell iron levels are precisely regulated. Ferritin, transferrin receptor (TfR) and ferroportin 1 (FPN1) play an important role in the management of iron metabolism. Accumulated evidence suggests that the transferrin–transferrin receptor (Tf–TfR) pathway might be the major route of iron transport across the luminal membrane of the capillary endothelium and iron uptake by nerve cells [17], [18]. In ischemic rat hearts, Tang et al [19] have reported that increased hypoxia inducible factor 1 (HIF-1) expression promotes the expression of TfR1 that leads to an increase in free iron. FPN1 [20], also known as IREG1 [21] or MTP1 [22], is a newly discovered transmembrane iron export protein. It is known that FPN1 is expressed on the basolateral surfaces of duodenal enterocytes and in macrophages, and plays a key role in iron transport across the basolateral membrane of enterocytes and in iron export during erythrocyte-iron recycling by macrophages [17]. Recent findings suggest that FPN1 is also expressed in the brain and might play a role in iron export from nerve cells [23], [24]. Most recently, Li et al. have shown that FPN1 mRNA expression was decreased in the cerebral cortex and hippocampus in rats subjected to cerebral ischemia [25]. However, the mechanisms responsible for increased free iron in an ischemic brain remain poorly understood.

Hepcidin, also known as ‘liver expressed antimicrobial peptide’ (LEAP), is an iron regulatory hormone mainly produced by hepatocytes in response to inflammatory stimuli, iron, and hypoxia [26], [27], [28], [29]. Functional studies have demonstrated that hepcidin binds to FPN1 in HEK 293 cells, leading to internalization of this complex and degradation of FPN1, thereby reducing cellular iron efflux [30]. The 19-amino acid peptide hepcidin-binding domain (HBD) of FPN1 has been identified by De Domenico et al. [31].

In the brain, iron homeostasis depends on both iron uptake by the cells and iron export from the cells [17]. TfR1 and divalent metal transporter 1 (DMT1) are important molecules responsible for iron uptake. FPN1 is the sole known cellular iron exporter and plays an important role in brain iron release. Recently, our laboratory found that hepcidin is widely expressed in the murine brain [32]. We further observed that, in the cerebral cortex, the hippocampus, and the striatum, hepcidin mRNA levels increased with aging. Injection of hepcidin into the lateral cerebral ventricle resulted in decreased FPN1 protein levels in the cerebral cortex, the hippocampus, and the striatum. Our results suggest that regulation of brain iron efflux by hepcidin could play a role in the control of brain iron metabolism. In the present study, experiments were carried out to test the hypothesis that hepcidin is involved in the iron overload in ischemia-reperfusion brain using rat and mouse stroke models of middle cerebral artery occlusion (MCAO). We also identified that increased TfR1 expression induced by HIF-1α may be another contributor to the iron overload in the ischemia-reperfusion brain.

## Materials and Methods

### Animals and experimental design

All procedures were carried out in accordance with the National Institutes of Health Guide for the Care and Use of Laboratory Animals, and were approved by the Animal Ethics Committee of Hebei Normal University. Wistar rats weighing 230–250 g and BALB/c mice weighing 20–25 g were given free access to food and water and kept in a 12 h light/dark cycle at 22.0±1.0°C. All rats and mice were allowed to adapt to their living conditions for at least 3 days before surgery.

The focal cerebral ischemia-reperfusion model of rats [33] and mice [34] was prepared according to a published method. Briefly, Wistar rats and BALB/c mice were anesthetized with 10% chloral hydrate (3.5 ml/kg, i.p.). After the left common carotid artery (CCA) was exposed through a midline neck incision, the CCA, external carotid artery (ECA), internal carotid artery (ICA) and pterygopalatine artery (PPA) were carefully separated from the adjacent tissue and vagus nerve. Then, two 5–0 silk sutures were tied loosely around the mobilized ECA stump and PPA. A 4-cm (for rats) and 2.5-cm (mice) length of fish wire (Beijing Sunbio Biotech, Beijing, China) was inserted through the incision of CCA into the ICA and thence into the circle of Willis, effectively occluding the MCA. The silk sutures around the ECA and PPA were lifted that is propitious to the suture reached to MCA. The silk suture around the CCA stump was then tightened around the intraluminal fish wire to prevent bleeding. The intraluminal occluders were made of fish wire and the diameter of the tip was 0.28 mm for rats and 0.18 mm for mice. After 1 h of MCAO, the fish wire was withdrawn from the ICA into the stump of the CCA and the withdrawal was stopped as soon as resistance was felt. The ischemic brain area starts the reperfusion. Then the animals were returned to their cages after recovering from anesthesia and allowed to survive for 24 h with free access to food and water.

The neurological score was assessed 24 h after reperfusion. The signs of neurologic impairment were evaluated according to Longa's 5-point scoring system 1 h after reperfusion (0 indicates no neurological symptoms; 1 indicates a failure to stretch the right forepaw completely; 2 indicates circling toward right; 3 indicates lateroversion toward the right when walking, and 4 indicates loss of walking). The score of all of the animals in the experimental group was 1, 2 or 3.

### Lateral cerebral ventricle injections

Hepcidin siRNAs or negative control siRNAs (Invitrogen, San Diego, CA) were suspended in RNase-free water to yield a concentration of 200 pmol/µL. 2.5 µL siRNA was combined with 2.5 µL tranxin and incubated at room temperature (RT) for 15 min. Mice were anesthetized with pentobarbital (40 mg/kg) and placed in a stereotactic device. Hepcidin or negative control (sham) siRNAs were slowly injected into the right lateral cerebral ventricle. Two hours later, the focal cerebral ischemia-reperfusion model was prepared as described above.

### Measurements of cerebral infarct

After neurological assessment, animals were sacrificed by decapitation. Intact brains were isolated and cut into 5 coronal sections. The brain slices were stained with 2,3,5-triphenyltetrazolium chloride (TTC) solution at 37°C for 30 min in the dark. After staining, normal brain tissue appeared as red, the circumjacent region of infarct as rose-pink, and the infarct region as white. The infarct tissues (ischemia side) and the normal tissues in the opposite side of the brain (control side) were collected for detection.

### Measurement of brain iron

The total iron in brain tissues was determined using Inductively Coupled Plasma Mass Spectroscopy (ICP-MS, Thermo Fisher, X Series, FL, USA). Before the experiments, all of the containers were soaked with 15% nitric acid for 24 h, washed with deionized water, and then rinsed with ultra-pure water before drying. Approximately 20 mg of brain tissues was added to 1 ml ultra-pure nitric acid (69.9–70.0%, J.T. Baker, USA) in Teflon digestion tubes, digested in the microwave digestion system for 2 h at 100°C, and then 4 h at 200°C [35]. The totally digested samples were diluted to 10 ml with ultra-pure water. Standard curves ranging from 0 to 100 ppb were prepared by diluting iron standard (1 mg iron/ml) with blanks prepared from homogenization reagents in 0.2% nitric acid. Standards and digested samples were read in triplicate by ICP-MS.

### Measurement of ferritin iron

Lysates of brain tissues were incubated for 10 min, cooled on ice, sonicated, and centrifuged at 12,000 rpm for 20 min, and the supernatant was collected. Protein concentrations were measured with a Nanodrop ND-1000 UV Spectrophotometer. A 200 µg portion was used for immunoprecipitation (IP). Samples were incubated at 4°C with a mixture of rabbit anti-H ferritin (Santa Cruz Biotechnology, CA, USA) and anti-L ferritin (Epitomics, CA, USA) antibodies for 3 h in order to precipitate the ferritins [36]. 40 µl protein A/G agrose (Santa Cruz Biotechnology, CA, USA) was added and incubated at 4°C for 12 h. The precipitate was separated by centrifugation and washed with TBS-T (50 mM Tris base, 150 mM NaCl, 0.1% SDS, 1% Triton X-100) buffer. Ultra-pure nitric acid (1 ml) was added and digested in the microwave digestion apparatus [37]. The total ferritin-bound iron was determined by ICP-MS.

### Reverse transcription (RT)-PCR

RNA was isolated from brain tissues by Trizol Reagent (Invitrogen, CA, USA). RT-PCR using total RNA (2 µg) was performed with the Revert AidTM First StrandcDNA Synthesis Kit (Takara, Dalian, China) according to the manufacturer's instructions. 1 µl cDNA was then used as a template for PCR. PCR amplification was performed with the following cycling parameters: denaturation at 94°C for 4 min, followed by 25 cycles (22 cycles for β-actin) at 94°C for 30 s, 60°C for 40 s, and 72°C for 1 min and then a single final extension at 72°C for 7 min. Expression of the target gene was determined by normalizing to the respective β-actin levels. Each amplification was repeated three times from different RT reactions, and the data were averaged. The following primers were used for PCR amplification, based on the cDNA sequence (GenBank accession number M26744.1, V01217 and AF344185): IL-6: Forward: 5′- TTCACAAGTCCGGAGAGGAG -3′; Reverse: 5′- GAGCATTGGAAGTTGGGGTA -3′. β-actin: Forward: 5′- GGTCACCCACACTGTGCC- CATCTA -3′; Reverse: 5′- GACCGTCAGGCAGCTCACATAGCTCT -3′; Hepcidin: Forward: 5′- CAA GAT GGC ACT AAG CAC TCG -3′; Reverse: 5′- GCT GGG GTA GGA CAG GAA TAA -3′.

### Western Blot analysis

Animal brains were perfused with cold saline. Brains were isolated on ice and homogenized in Lysis buffer (0.5% phenyl methylsulfonyl fluoride, 0.1% Pepstatin, 0.1% Leupeptin, 0.1% Aprotinin in TBS-T). After centrifugation at 10,000 g for 30 min at 4°C, the supernatant was collected and protein concentration was measured with a Pierce BCA protein assay kit (Thermo Scientific, Rockford, IL, USA). Aliquots of the extract containing 50 µg of protein were separated by reducing SDS-PAGE (10% for FPN1, TfR1, HIF1α and STAT3, 12% for L-ferritin and H-ferritin), and electroblotted onto nitrocellulose filter membranes for 45 min at RT. The membranes were blocked in 5% non-fat milk containing 20 mM Tris–HCl, pH 7.6, 137 mM NaCl, 0.1% Tween-20 (TBS-T) for 2 h at room temperature, and then incubated with antibodies against L-ferritin (1∶5000), H-ferritin (1∶2000), FPN1 (1∶5000) (Alpha Diagnostic International, San Antonio, TX), TfR1 (1∶2000) (Zymed Laboratories; South San Francisco, CA), HIF-1α, STAT3, p-STAT3 (1∶2000) (Sigma-Aldrich, St. Louis, MO) overnight at 4°C. After washing with TBS-T three times, the blots were incubated in secondary-antibody-conjugated horseradish peroxide (1∶5000) (Amersham, UK) for 2 h at room temperature. Immunoreactive proteins were detected by using the enhanced chemiluminescence method (ECL kit, Amersham, UK), and quantified by transmittance densitometry using volume integration with Gel-Pro software. To ensure even loading of the samples, the same membrane was probed with rabbit anti-human β-actin antibody (1∶5000) (Sigma-Aldrich, St. Louis, MO).

### Immunohistochemistry

For immunohistochemical analysis, animals were anesthetized with 1% pentobarbital sodium (40 mg/kg body weight, i.p.), perfused with saline, followed by 4% paraformaldehyde in 0.1 mol/L phosphate buffered solution (PB), pH 7.2. Brains were removed and fixed in 4% paraformaldehyde for 4–6 h at 4°C. They were then immersed in 30% sucrose in 0.1 mol/L PB solution for 1–2 days. Serial sections (15 µm) were cut using a cryostat (Leica CM1900, Germany) and mounted on slides. Prior to staining, sections were treated with 3% hydrogen peroxide followed by washing with PB and incubation with 10% normal goat serum in PB for 1 h at RT. Primary rabbit anti-mouse hepcidin (1∶500 dilution, Alpha Diagnostic International, San Antonio, TX) was added and incubated overnight at 4°C. After washed 3 times with PB for 10 min, the brain sections were incubated with biotinylated goat anti-rabbit secondary antibody (Zymed Laboratories; South San Francisco, CA, 1∶200) for 60 min at 37°C. After washing, the sections were treated with streptavidin–horseradish peroxidase conjugate (1∶200 dilution; Zymed Laboratories) for 60 min at 37°C. The slides were then washed four times with PB for 5 min and the staining reaction was carried out using 3,3′-diaminobenzidine tetrahydrochloride (DAB) as the chromogen. Negative controls were processed by replacing the primary antibody with diluent rabbit serum. The sections were dehydrated in ethanol, cleared in xylene, and cover-slipped with neutral balsam. Finally, slices were photographed with Zeiss Imager A2 microscope (Germany).The same areas were selected for imaging in cortex, in hippocampus and in corpus striatum. The photographs were analyzed with the Image-Pro Plus software, and the AOI was set as the whole image.

The intensity was calibrated with Std. Optical Density (OD). Manual color was selected (in the count/size column); and the background Gray Level was set at 150 in all slices, and count 150 to 255 (max) signal in the histogram. Third, the positive Area and Density (mean) were selected. Finally, we counted the positive area to get the mean Density.

### Statistical Analysis

All quantitative data are presented as mean ± SEM. Differences between means in two groups were carried out by unpaired-samples T-test. A probability value of P<0.05 was considered to be statistically significant.

## Results

### Increased ferritin levels in the cerebral cortex, hippocampus, and corpus striatum

**Figure 1:**
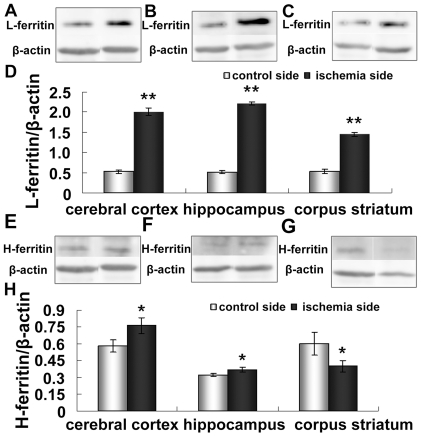
Expression of L-ferritin and H-ferritin in ischemic brain. Rats were subjected to 60 min MCAO and 24 hour reperfusion. Contralateral and ipsilateral cerebral cortex (A,E), hippocampus (B,F) and corpus striatum (C,G) were isolated, homogenized, and analyzed for L- and H- ferritin by Western blotting. Representative ferritin blots and corresponding β-actin blots are shown on the top panel. The lower panel (D, H) shows the summary of L-ferritin and H-ferritin expression levels from three animals in each group. Results are presented as Mean ± SEM, n = 3. **p<0.01 v.s. contraleteral side, *p<0.05 v.s. contralateral side.

Ferritin is a ubiquitous and highly conserved iron binding protein, which consists of two subunits (H and L), and it is a major form of non-heme iron stores in cells. We performed western blot analyses to determine the ferritin protein levels in different regions of an ischemic brain. Fig. 1 shows the ferritin levels in cerebral cortex, hippocampus, and corpus striatum of both control and ischemia sides. Compared with the control side, the level of L–ferritin increased significantly in the three regions on the ischemia side (p <0.01) (Fig.1D). Similarly, the H-ferritin protein level was also significantly elevated in the cerebral cortex and the hippocampus but decreased in the corpus striatum (Fig. 1H) (p <0.05).

### Decreased FPN1 levels in the cerebral cortex, hippocampus, and corpus striatum

**Figure 2:**
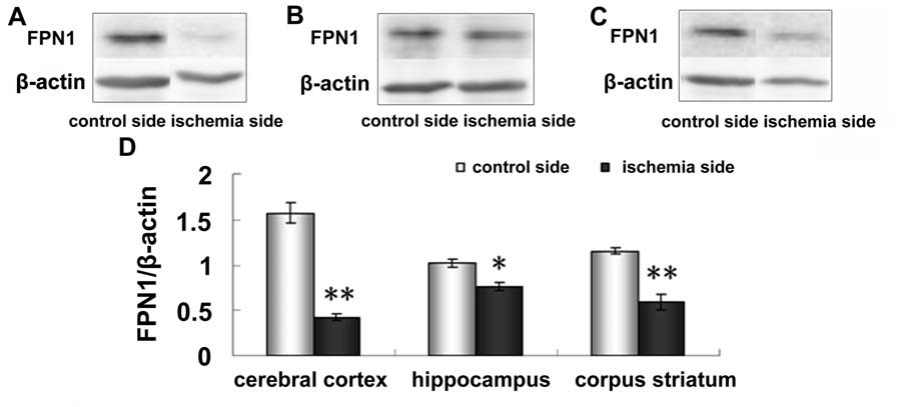
Expression of FPN1 in ischemic brain. Rats were subjected to 60 min MCAO and 24 hour reperfusion. The expression of FPN1 in the cerebral cortex (A), hippocampus (B) and corpus striatum (C) of contralateral and ipsilateral side was analyzed by Western blotting. Representative bands from contralateral side and ipsilateral side are shown on the top panel. The lower panel (D) shows the summary of FPN1 expression from three animals in each group. The expression of FPN1 was very significantly increased in the ischemic cerebral cortex, corpus striatum at 24 h, and was significantly increased in the ischemic hippocampus at 24 h. Results are presented as Mean ± SEM, n = 3. **p<0.01 v.s. contraleteral side, *p<0.05 v.s. contralateral side.

FPN1 is a unique protein involved in iron efflux. Our previous study elucidated that FPN1 increases iron release in PC12 cells [23] and primary cultured neurons [23], [32]. The present results demonstrate that FPN1 protein levels in the cerebral cortex (Fig.2A) and corpus striatum (Fig.2C) significantly decreased in the ischemia side compared with the control side (p <0.01) (Fig.2D). In addition, the expression of FPN1 in the hippocampus (Fig.2B) also decreased significantly in the ischemia side (p <0.05) (Fig.2D). These results indicate that ischemia may increase intracellular iron levels by reducing FPN1 expression.

### Increased expression of hepcidin in the cerebral cortex, hippocampus, and corpus striatum

**Figure 3:**
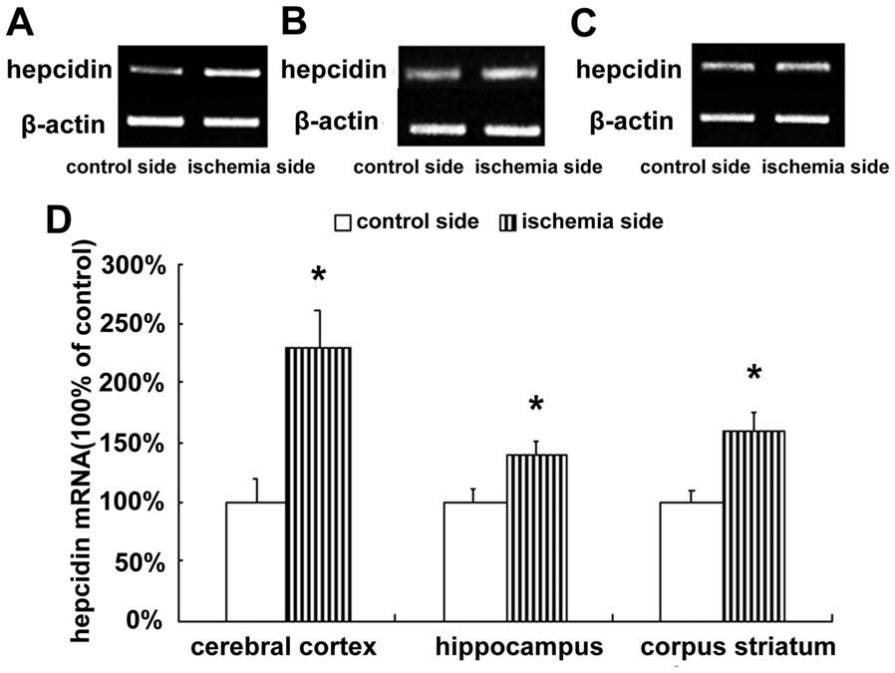
mRNA expression of hepcidin in ischemic brain. Rats were subjected to 60 min MCAO and 24 hour reperfusion. RT-PCR analyses were carried out to evaluate the mRNA level of hepcidin in the cerebral cortex (A), hippocampus (B) and corpus striatum (C) of contralateral and ipsilateral side. Fig. 3D shows the summary of hepcidin expression from three animals in each group. The expression of hepcidin was significantly increased in the ischemic cerebral cortex, hippocampus and corpus striatum at 24 h. Results are presented as Mean ± SEM, n = 3. *p<0.05 v.s. contralateral side.

**Figure 4:**
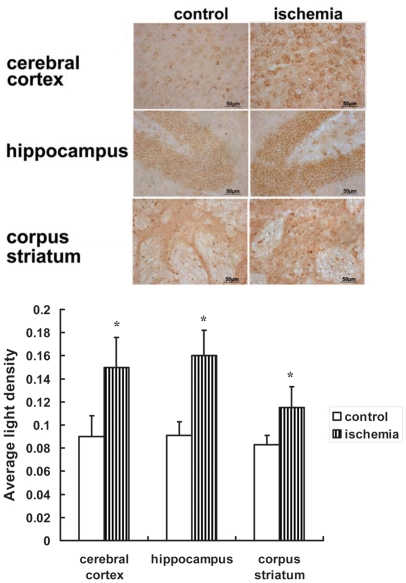
Positive hepcidin/pro-hepcidin staining in ischemic brain. Hepcidin/pro-hepcidin was detected by immunohistochemistry. Hepcidin/pro-hepcidin positive staining was significantly increased in cerebral cortex, hippocampus and corpus striatum of ischemic brain. The mean density of positive staining was measured by Image-Pro Plus soft. Data are presented as means ±SEM, n = 3. *p<0.05, vs. contralateral side.

Hepcidin regulates iron efflux by binding to FPN1 and inducing its internalization [38]. To investigate the mechanism of FPN1 down-regulation, we examined the mRNA expression of hepcidin in ischemic brain tissue. The results show that the hepcidin mRNA level (Fig.3) and hepcidin/pro-hepcidin protein levels (Fig. 4) were significantly up-regulated in the cerebral cortex, hippocampus, and corpus striatum of the ischemic hemisphere.

### Increased in FPN1 and decreased L-ferritin protein level by knocking down hepcidin

**Figure 5:**
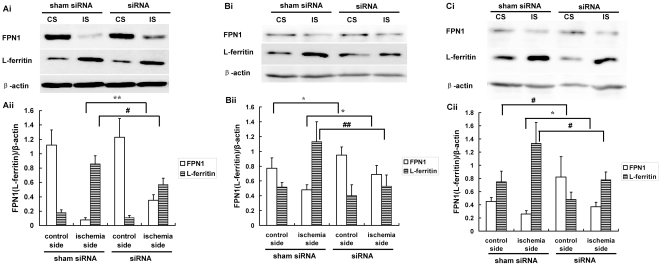
Expression of FPN1 and L-ferritin protein levels in the cerebral cortex, hippocampus and corpus striatum of mouse after hepcidin siRNA injection in the lateral cerebral ventricle. A representative Western blot of the FPN1 and L-ferritin was presented. Quantification of expression of PFN1 and L-ferritin protein in the cerebral cortex (A), hippocampus (B) and corpus striatum (C) of mouse. Expression values were normalized for β-actin. The data were presented as means ± SEM, n = 3. *p<0.05, **p<0.01 vs. FPN1 of ischemic side (IS) or control side (CS), #p<0.05, ##p<0.01 v.s. L-ferritin of ischemic side (IS) or control side (CS). Sham siRNA (negative control treated group), siRNA (specific hepcidin siRNA treated group).

The above results implied that the up-regulated hepcidin expression may be an important factor that down-regulates the FPN1 and further increases the iron content in an ischemic brain. In order to confirm the reuslt, we determined the effect of a specific hepcidin siRNA on the FPN1 and L-ferritin levels. The hepcidin siRNA treatment increased the FPN1 level in cerebral cortex (Fig. 5A, p<0.01), hippocampus (Fig. 5B, p<0.05), and corpus striatum (Fig. 5C, p<0.05) of the ischemic side of the mouse brains. We next determined the effect of hepcidin siRNA on the L-ferritin level in the ischemic barin. Results from Western blot analysis demonstrated that hepcidin siRNA treatment rescued the L-ferritin increase in the cerebral cortex (Fig. 5A, p<0.05), hippocampus (Fig. 5B, p<0.01) and corpus striatum (Fig. 5C, p<0.05). The repressing pattern of L-ferritin was negatively correlated with the FPN1 increasing pattern. These results further proved that hepcidin is involved in the iron regulation in the ischemic brain.

### Increased Expression of IL-6 in the cerebral cortex, hippocampus and corpus striatum

**Figure 6:**
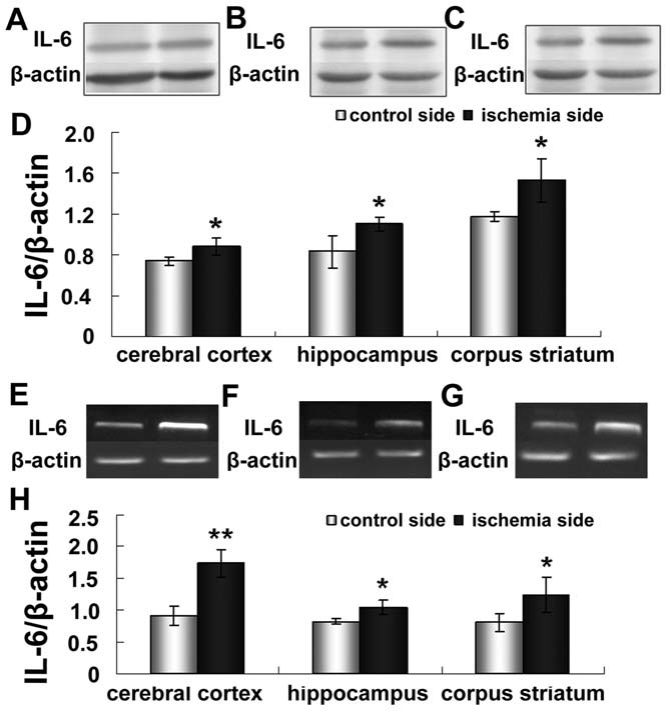
Expression of IL-6 mRNA and protein in ischemic brain. Rats were subjected to 60 min MCAO and 24 hour reperfusion. The IL-6 protein level of cerebral cortex (A), hippocampus (B) and corpus striatum (C) were analyzed Western blotting. The mRNA level of cerebral cortex (E), hippocampus (F) and corpus striatum (G) of contralateral and ipsilateral side was detected by RT-PCR. Fig 4D and Fig 4H show the summary of IL-6 protein and mRNA expression respectively. Results are presented as Mean ± SEM, n = 3. **p<0.01 v.s. contraleteral side, *p<0.05 v.s. contralateral side.

The inflammatory cytokine interleukin-6 (IL-6) directly regulates hepcidin through induction and subsequent promoter binding of signal transducer and activator of transcription 3 (STAT3) [39], [40]. STAT3 is necessary and sufficient for the IL-6 responsiveness of the hepcidin promoter[41]. To understand the mechanism of ischemia-induced upregulation of hepcidin, we studied both IL-6 protein and mRNA expression (Fig.6). The expression of IL-6 protein significantly increased in the ipsilateral cortex, the hippocampus and the corpus striatum (p <0.05) (Fig.6D). The mRNA levels of IL-6 were significantly elevated in the cortex on the ischemic side (p <0.01 vs. control cortex), the hippocampus, and the corpus striatum (p <0.05 vs. the control hippocampus and corpus striatum) (Fig.6H).

**Figure 7:**
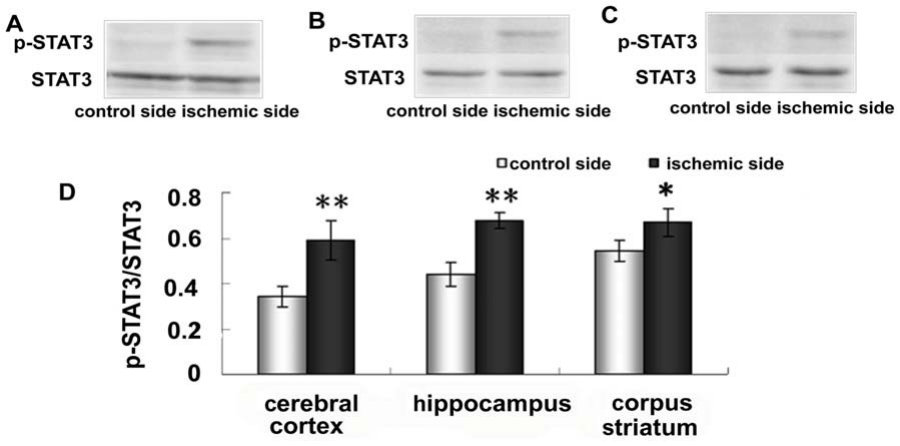
Expression of STAT3 in ischemic brain. Rats were subjected to 60 min MCAO and 24 hour reperfusion. Expression of STAT3 in cerebral cortex (A), hippocampus (B) and corpus striatum (C) was analyzed by Western blotting. D, summary of STAT3 expression in cerebral cortex hippocampus, and corpus striatum of contralateral and ipsilateral side. Results are presented as Mean ± SEM, n = 3. **p<0.01 v.s. contraleteral side, *p<0.05 v.s. contralateral side.

To understand the mechanism of upregulation of hepcidin caused by brain ischemia, we further examined the expression of STAT3 and phosphorylated STAT3 (p-STAT3) in the cerebral cortex (Fig.7A), hippocampus (Fig.7B) and corpus striatum (Fig.7C). As shown in Fig. 7, the ratio of p-STAT3 to STAT3 on the ischemia side was much higher than on the control side in the cerebral cortex (p <0.01), hippocampus (p <0.01) and corpus striatum (p <0.05) (Fig.7D).

### Increased TfR1 protein levels in the cerebral cortex and hippocampus

**Figure 8:**
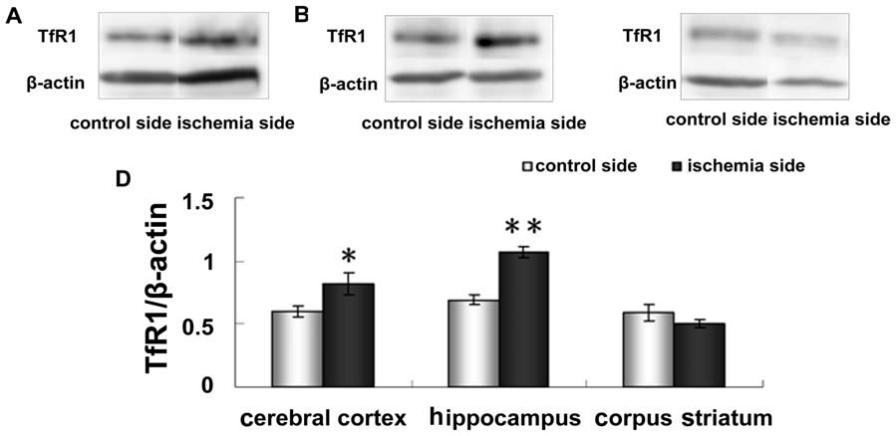
Expression of TfR1 in cerebral cortex (A), hippocampus (B) and corpus striatum (C) of contralateral and ipsilateral side by western blot analysis. Representative TfR blots and corresponding β-actin blots are shown on the top panel. The lower panel (D) shows the summary of TfR expression from three animals in each group. Results are presented as Mean ± SEM, n = 3. **p<0.01 v.s. contraleteral side, *p<0.05 v.s. contralateral side.

TfR1 is a key protein that facilitates iron transport into brain cells. We observed that the TfR1 protein level was dramatically elevated in the cerebral cortex (Fig. 8A, 8D, p <0.05) and hippocampus (Fig.8B, p <0.01) on the ischemic side (Fig.8D), compared to their control counterparts. However, there was no significant change in TfR1 levels in the corpus striatum between the ischemic and control sides (Fig. 8C and 8D).

### Up-regulation of HIF-1α protein in the cerebral cortex, hippocampus and corpus striatum

**Figure 9:**
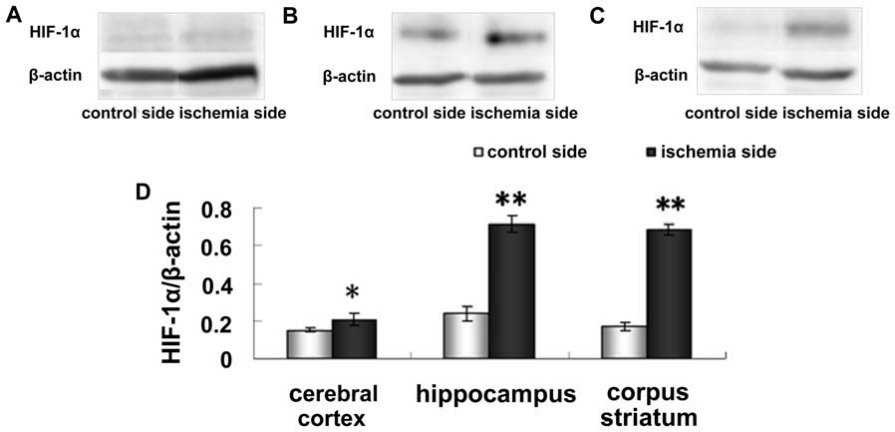
Western blot analysis shows the expression of HIF-1α in cerebral cortex (A), hippocampus (B) and corpus striatum (C) of contralateral and ipsilateral side. Representative HIF-1α bands and corresponding β-actin bands are shown on the top panel. The lower panel (D) shows the summary of HIF-1α expression from three animals in each group. Results are presented as Mean ± SEM, n = 3. **p<0.01 v.s. contraleteral side, *p<0.05 v.s. contralateral side.

HIF-1 is a key regulator in hypoxia, and has been suggested as an important player in ischemic stroke due to the functions of its downstream genes that promote glucose metabolism, angiogenesis, erythropoiesis, and cell survival. In our results, the expression of HIF-1α in the ischemia side of the cerebral cortex (Fig.9A) was significantly increased compared with the control side (p <0.05) (Fig.9D), and the expression of HIF-1α in the ischemia side of the hippocampus (Fig.9B) and corpus striatum (Fig.9C) were significantly increased compared with the control side (p <0.01) (Fig.9D).

## Discussion

Ischemic stroke occurs when the blood supply to the brain is suddenly interrupted by a blood clot blocking a blood vessel in the brain, and is a major cause of disability. There has been very limited progress in developing effective therapeutic approaches for ischemic stroke, although numerous agents have been tested in animal models and in clinical trials. Among many factors, incomplete understanding of the mechanism responsible for ischemia-caused neuronal injuries is a major hurdle. Cerebral ischemia induces a series of molecular pathways involving signaling mechanisms, gene transcription, and protein modification. Free radicals and oxidative stress have been suggested to be involved in each of the steps in the injury cascade [42]. Iron is the most important metal element that mediates free radical generation via a Fenton-type reaction. Furthermore, hypoxia and free iron appear to interact with each other in causing subsequent neuronal death [13].

**Figure 10:**
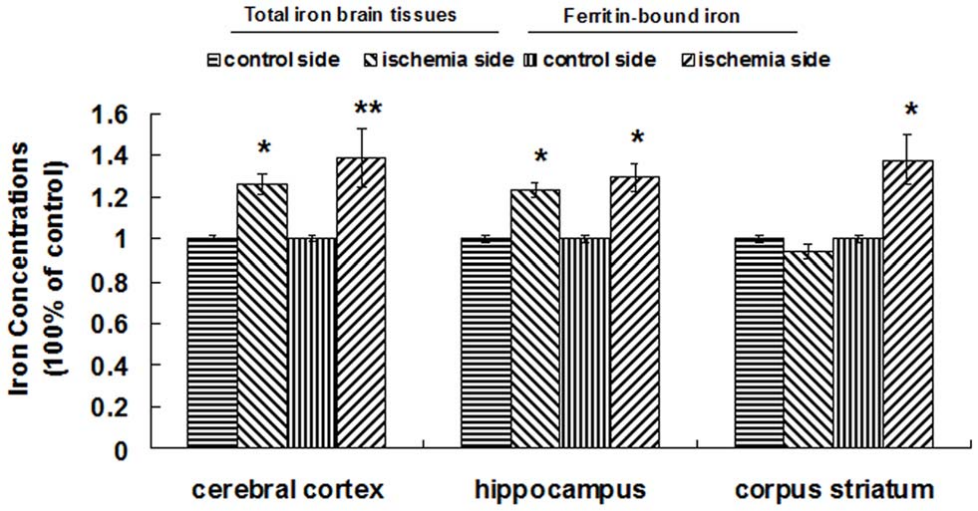
Effect of ischemia/reperfusion on the total iron and ferritin binding iron in the brain. Total iron was masured by ICP-MS, the ferritin bound iron was detected firstly by Immunoprecipitation and ICP-MS. The first two panels in the 3 groups represent the relative total iron concentrations in brain tissues (per milligram of wet brain tissue). The last two panels of 3 groups represent the ferritin-bound iron (per milligram of total protein). The data were presented as mean ± SEM (100% of control), n = 6, *P<0.05. **P<0.01.

Our present results demonstrate that the iron-storage proteins L-ferritin and H-ferritin are remarkably elevated in the cerebral cortex and the hippocampus. Previous studies have shown that ischemic preconditioning initiates synthesis of ferritin in the heart [37] and retina [36]. It has been proposed that this increase in ferritin might protect cells from iron-mediated oxidative damage during ischemia-reperfusion. Our present data show that L-ferritin and H-ferritin are increased in ischemic cerebral cortex and hippocampus (Fig.1), and may provide a mechanism to reduce iron-mediated injury by reducing free iron. Our results reveal that both total iron and ferritin-bound iron are significant increased in the ischemia side of the cerebral cortex and hippocampus (Fig. 10). Although we cannot conclude that ferritin plays a protective role based on these results, the increased levels of ferritin indicate that free iron levels in these tissues should have been reduced by binding.

Functional studies have demonstrated that hepcidin is the central regulator of systemic iron homeostasis by regulating FPN1, the only protein known to release iron from cells [43]. Recently, data from our laboratory and others have shown that hepcidin is widely expressed in murine brain and has a very important role in regulating iron levels in the brain by down-regulating FPN1 expression [44], [45]. However, the role of hepcidin has not been studied in the ischemic brain. The present study demonstrates that ischemia-reperfusion increases hepcidin expression and down-regulates FPN1 protein expression in the cerebral cortex and the hippocampus. Knockdown hepcidin reversed the FPN1 decrease and ferritin increase in the ischemic brains of mice. Our present results demonstrated that down-regulated expression of FPN1 by hepcidin may be one important mechanism responsible for the ischemia-induced iron increase in the brain. In other words, reduced iron release from ischemic brain cells due to down-regulation of FPN1 increases intracellular iron levels [46].

It is known that inflammation up-regulates hepcidin expression and that cytokine IL-6 is a major mediator of the inflammatory response. It has been reported that IL-6 leads to increase in hepcidin production and decrease in serum iron within a few hours in humans and experimental animals [39], [47]. Previous studies have revealed the mechanism responsible for IL-6 induced hepcidin expression [41], [48]. Briefly, IL-6 can activate the JAK/STAT signaling pathway that upregulates the level of p-STAT3. p-STAT3 binds to the *HAMP* promoter and thus increases hepcidin expression. It is well established that inflammation is involved in stroke and that ischemia increases IL-6 concentrations in brain tissues. Indeed, in our ischemic model we observed that cerebral ischemia-reperfusion increased IL-6 production significantly. Furthermore, the ratio of p-STAT3/STAT3 in ischemic tissues was much higher than in control tissues. This implies that the increased hepcidin in ischemic tissues may be regulated through the JAK-STAT pathway.

HIF-1 is one of the factors activated in early ischemia that can induce vascular endothelial growth factor, erythropoietin, etc. [49]. Those downstream genes of HIF-1 promote cell survival by generating new blood vessels and providing new oxygen supply channels. HIF-1 is composed of HIF-1α and HIF-1β protein subunits. HIF-1β is constitutively expressed and relatively stable. The HIF-1α level is dependent on tissue oxygen levels and primarily determines HIF-1 activation. Tang et al. have studied the relationship between HIF-1α and transferrin. They have observed that the activation of HIF-1α can induce TfR1 expression, which, in turn, enhances iron accumulation and eventually increases oxidative damage and lipid peroxidation [50]. Moreover, previous studies have clearly shown that deferoxamine, an iron chelator, can reduce injury caused by cardiac ischemia and reperfusion [50] and cerebral ischemia [51]. In the present study, we observed that the HIF-1α expression level is increased in the ischemic brain, and this result is in agreement with previous reports [52]. The increased HIF-1α may play a key role in up-regulating TfR1 expression in the cerebral cortex and hippocampus of the ischemic brain. Thus, the increased TfR1 may be another pathway by which the iron and ferritin levels are elevated in ischemic brain cells. The sources of the iron might be extracellular fluid, which is potentially involved in both intracellular iron storage and intravascular iron transport and delivery to the brain [9].

Surprisingly, we did not observe increased total iron and H-ferritin in ischemic corpus striatum. The proportion of total iron and ferritin-bound iron in ischemic and control areas was different in the cerebral cortex, the hippocampus, and the corpus striatum (Fig. 10). These results imply that the ischemia-induced iron change is region-dependent. The mechanisms of the area-dependent iron changes are likely complex although the hepcidin-FPN1 and Tf-TFR pathways may play a role.

**Figure 11:**
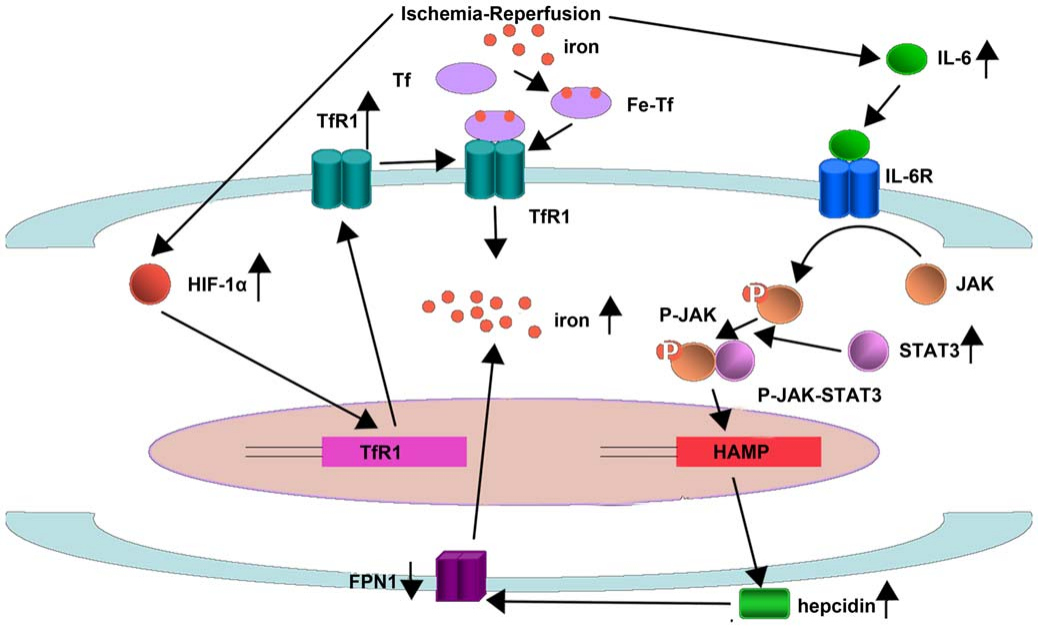
Proposed pathways for the regulation of the iron level in cerebal ischemia. Tf: Transferrin; TfR1: Transferrin receptor; TfR1: gene of Transferrin receptor; HIF-1α: Hypoxia-inducible factor-1α; FPN1: Ferroportin1; IL-6: Interleukin-6; IL-6R: Interleukin-6 receptor; HAMP: gene of hepcidin; STAT3: Signal transducing activator of transcription-3.

In summary, the current observations revealed two pathways that contribute to iron overload in ischemic brain tissues as outlined in Figure 11. First, ischemia-reperfusion increases the expression of IL-6 that up-regulates hepcidin by the JAK/STAT3 pathway. Hepcidin induces FPN1 internalized degradation, reduces the efflux of iron, and thus causes iron accumulation. Second, ischemia-reperfusion up-regulates the HIF-1α level, which upregulates TfR1 expression and accelerates iron accumulation in the ischemic tissues.
